# *BCL11B*-related disease: a single phenotypic entity?

**DOI:** 10.1038/s41431-025-01824-x

**Published:** 2025-03-03

**Authors:** J. Heather Vedovato-dos-Santos, Rebecca S. Tooze, Sivagamy Sithambaram, Emma McCann, Yasemin Alanay, Ozlem A. Dogan, Meltem Kilercik, Aysen Bingol, Memet M. Ozek, David Johnson, Christoffer Nellaker, Andrew O. M. Wilkie, Stephen R. F. Twigg

**Affiliations:** 1 Clinical Genetics Group, MRC Weatherall Institute of Molecular Medicine, University of Oxford, Oxford, UK; 2 Jesus College, Oxford, UK; 3 Department of Clinical Genetics, Liverpool Women’s NHS Foundation Trust, Liverpool, UK; 4 Division of Pediatric Genetics, Department of Pediatrics, Acibadem University, School of Medicine, Istanbul, Turkey; 5 Rare Diseases and Orphan Drugs Application and Research Center-ACURARE, Acibadem University, Istanbul, Turkey; 6 Division of Medical Biochemistry, Department Of Basic Sciences, Acibadem University, School Of Medicine, Istanbul, Turkey; 7 Division of Pediatric Allergy and Immunology, Department of Pediatrics, Akdeniz University, School of Medicine, Antalya, Turkey; 8 Department of Neurosurgery, Acibadem University, School of Medicine, Istanbul, Turkey; 9 Oxford Craniofacial Unit, Oxford University Hospitals NHS Foundation Trust, Oxford, UK; 10 Big Data Institute, Nuffield Department of Women’s & Reproductive Health (NDWRH), University of Oxford, Oxford, UK; 11 NIHR Oxford Biomedical Research Centre, Oxford, UK

**Keywords:** Disease genetics, Medical genetics

## Abstract

Craniosynostosis (CRS), the premature fusion of sutures between the skull bones, is characterised by a long “tail” of rare genetic diagnoses. This means that pathogenic variants in many genes are responsible for a minority of cases, and identifying these disease genes and delineating the associated phenotype is extremely important for patient diagnosis and for genetic counselling of families. One such gene is *BCL11B*. Heterozygous pathogenic variants in *BCL11B* have been described as causative for two Mendelian phenotypes, but until recently the gene remained only marginally associated with CRS. We have carried out a systematic review of literature, providing evidence that *BCL11B*-related disease (BRD) should be regarded as a single phenotypic entity. Furthermore, we describe four new patients, all of whom presented with CRS, thus expanding the phenotype of BRD and highlighting CRS as an important diagnostic clue.

## Introduction

Craniosynostosis (CRS) describes the premature fusion of one or more of the sutures separating the cranial bones [1]. It has a prevalence of between 1 in 1400 and 1 in 2100 children and occurs in association with other syndromic features in approximately 30% of cases [2, 3]. CRS is aetiologically heterogeneous. Approximately 84% of the monogenic component can be screened by testing seven main genes, and the remaining 16% of cases are caused by variants in over fifty genes, each of which is responsible for a small number of cases [4]*. BCL11B* (*BAF Chromatin Remodelling Complex Subunit BCL11B*, chromosome 14q32.2) is one such gene.

*BCL11B* is a highly conserved gene that encodes a C_2_H_2_-type zinc finger transcription factor of 894 amino acids (NM_138576.4) [5]. BCL11B is regulated by the NURD (nucleosome remodelling and histone deacetylase) complex and has been shown to be important for the development of T-cells and various neuronal subtypes [6–9]. Heterozygous pathogenic variants in *BCL11B* have been described as causative for two Mendelian disorders: Severe combined immune deficiency, 49 (IMD49; OMIM: #617237) [10]; and Intellectual developmental disorder with dysmorphic facies, speech delay and T-cell abnormalities (IDDSFTA; OMIM: #618092) [11]. Craniosynostosis was absent from the original descriptions of these syndromes. Since then, however, the phenotype and the spectrum of pathogenic variants associated with *BCL11B*-related disease (BRD) have expanded as the publication rate of case reports went from 2 papers/year from 2019–2021 [12–17] to 5.5 papers/year in 2022–2023 [18–28]. Furthermore, Clinvar currently (July, 2024) lists 50 unique pathogenic/likely pathogenic variants (not counting large deletions/duplications), more than 30% of which have been added since 2022.

Here we describe four additional individuals with pathogenic variants in *BCL11B*, including the first instance of a mosaic pathogenic variant in a proband. All four patients presented with CRS, thereby increasing the number of BRD patients reported with this clinical finding and highlighting it as an important diagnostic feature. Our comprehensive review of the literature indicates the absence of a significant distinction between IMD49 and IDDSFTA, either in terms of clinical presentation or molecular mechanism. Finally, we show that *BCL11B* has been underappreciated as a contributor to the phenotype resulting from 14q32 microdeletion and that where BRD is suspected, targeted copy number analysis should be undertaken after negative sequencing results.

## Methods

### Case reports

Patients were identified through data from the Genomics England 100,000 Genomes Project (Patient 1) [4, 29], an international collaboration (Patient 2) and the Oxford Craniofacial Unit (Patients 3 and 4). Routine genetic testing included sequencing of mutation hotspots in *FGFR1*, *FGFR2* and *FGFR3*, complete sequencing of *TWIST1* and *TCF12*, array comparative genomic hybridisation (CGH), trio exome sequencing (Patient 2) and genome sequencing (GS) (Patient 4). The mosaic variant in Patient 1 was missed in the routine clinical analysis and detected later through repeated bioinformatics analysis [4] and confirmed by deep sequencing of *BCL11B* (methods described below).

#### DNA extraction for deep sequencing

DNA was extracted from patient samples (blood and saliva) using protocols and reagents detailed in the Zymo Midiprep Kit (Zymo - Orange, CA, USA). Saliva (2 ml) was mixed with an equal volume of BioFluid and Cell Buffer and 70 μl of proteinase K (20 mg/ml). Samples were mixed thoroughly before incubation at 55 °C for 2 h. DNA was quantified using the Qubit dsDNA broad range assay (Q32850, Life Technologies) and the quality was assessed by gel electrophoresis.

#### Deep sequencing

DNA extracted from blood and saliva samples was analysed by next-generation sequencing (MiSeq, Illumina). Primers containing CS tags were used to amplify a 213 bp fragment of *BCL11B*: forward (5’: ACACTGACGACATGGTTCTACAGATGAGCCTTCCAGCTACATT), reverse (5’: TACGGTAGCAGAGACTTGGTCTCTGTCGCCCAGGAAATTCA). A 100 ng sample of genomic DNA was amplified using a Q5 (New England Biolabs - Ipswich, MA, USA) protocol (2 μl of 5x Q5 buffer, 0.5 μl of each forward and reverse primer containing CS tags (10 μM), 0.2 μl of dNTPs (10 mM), 0.1 μl of Q5 high-fidelity DNA polymerase in 10 μl) and thermocycler conditions: 98 °C for 30 s, followed by 30 cycles of 98 °C for 10 s, 66–70 °C for 30 s, and 72 °C for 30 s, and a final extension of 72 °C for 8 min. PCR products were diluted in nuclease-free water 1:100 before barcoding. Diluted products (1 μl) were mixed with 5 μl of 2x iProof High-Fidelity Master Mix (BIO-RAD – Hercules, CA, USA), 2 μl of water, and 2 μl of Fluidigm Access Array CS barcodes (2 μM) and placed in the thermocycler: 98 °C for 2 min, followed by 8 cycles of 98 °C for 10 s, 60 °C for 30 s, and 72 °C for 30 s, and a final extension of 72 °C for 2 min. Barcoded products were pooled and quantified (Qubit dsDNA High Sensitivity protocol). The purified pool was checked using a 2200 TapeStation instrument (Agilent High Sensitivity D1000 ScreenTape) prior to sequencing using MiSeq. Data were analysed, including alignment, variant calling, and annotation using amplimap software [30] (Supplementary Material F1–F3).

#### Variant classification

Sequence variants (Patients 1, 2 and 4) were analysed and classified according to the American College of Medical Genetics (ACMG) guidelines of 2015 [31]. The deletion in patient 3 was analysed according to the ACMG guidelines of 2020 [32]. Table 1 summarizes the variants, their classification and the criteria used to determine pathogenicity.

**Table 1:** Clinical and molecular features in newly reported patients.

	Patient 1	Patient 2	Patient 3	Patient 4
Sex	Female	Male	Female	Female
Age (years, months)	9 yr 5 mo	3 yr 4 mo	14 yr 6 mo	4 yr
Variant	c.781_808dup; p.(Gly270Alafs*256)	c.2438_2459del; p.(Val813Alafs*24)	arr[hg19]14q32.2(99,052,763–100,591,634)x1^a^	c.400_403dup; p.(Cys135Tyrfs*61)
De novo variant?	+ (mosaic)	+	Not tested	+
ACMG classification	Pathogenic (PVS1 + PS2 + PM1 + PM2)	Pathogenic (PVS1 + PM1 + PM2)	Pathogenic (1A + 2A + 3A = 1.00)	Pathogenic (PVS1 + PM2 + PM6)
Thin/Sparse eyebrows	+	+	+	+
Small palpebral fissures	−	−	+	−
Apparent hypertelorism	−	−	−	+
Long/smooth philtrum	+	+	+	+
Thin upper lip vermilion	−	+	+	+
Intellectual disability	+	+	+	+
Speech impairment	+	+	+	+
Delayed motor milestones	+	+	+	+
Autistic features	−	N/A	+	−
Frequent/atypical infections	−	+	−	−
Laboratory immune anomalies	−	+	N/A	+
Allergy/Asthma	−	+	−	+
Refractive error	+	−	+	−
Dental anomalies	+	−	−	N/A
Feeding difficulties	−	−	+	−
Brain MRI abnormality	−	−	N/A	N/A
Craniosynostosis	+	+	+	+

+ present, − absent, *N/A* information not available.

^a^NC_000014.8:g.99052764_100591635del.

### Literature review

The literature search was conducted from November 2022 to March 2023. Initially, two databases were searched (MEDLINE and Embase) with the keyword [BCL11B] in titles and abstracts. This rendered 444 and 656 results on each database respectively, all of which were screened against a single inclusion criterion: mention of a patient with a germline pathogenic/likely pathogenic variant in *BCL11B*. A snowballing approach was taken on all selected results (backward via reference lists). Redundant searches were conducted to make sure that no relevant papers were missed, using [BCL11B] as the first concept and one of the following terms as the second concept: [craniosynostosis], [craniosynostoses], [suture*], [crani*]. We also returned to the literature periodically, for publications that emerged during the writing of this paper.

A limitation was the availability of clinical information in the papers included in our review; we could not be sure whether the definitions of other authors were the same as ours, and it was not always clear whether a given clinical feature was absent or not assessed. To minimize the impact of these issues, when calculating percentages, we disregarded patients for whom a specific feature was not assessed or for whom there was no information available. As a rule, if a specific feature was not mentioned, we did not assume absence, characterising it instead as N/A (“information not available”) (Table 1; Supplementary Table 1). We identified several clinical features which seem to have a high prevalence in patients with BRD, but that have not been systematically assessed in previous cohorts (craniosynostosis, behavioural anomalies, sleeping problems, hypotonia, constipation and/or reflux disease and dermatitis/eczema). For these features, when calculating percentages, the total reflects the total number of patients for whom there is a substantial description of the phenotype, regardless of whether the specific feature was mentioned as present or absent. Therefore, the prevalence of these features may be underestimated.

To search for whole gene deletions of *BCL11B* or 14q32 microdeletions resulting in BRD, we queried the same databases with appropriate keywords and mapped subject headings in titles and abstracts (Supplementary Material M1-M2). This rendered 403 results on Medline and 659 results on Embase. An arbitrary limit of 5 Mb was chosen and deletions larger than that or those patients for whom breakpoint coordinates and clinical information were not available were excluded. All results were screened against the inclusion criteria described in the Supplementary Material. We compared the clinical features of the *BCL11B* gene deletion cohort with two distinct sets of clinical features: those associated with BRD and those associated with 14q32-qter microdeletions [33] (Supplementary Material M5).

### Analysis of the facial phenotype

Where facial photographs were available, we used our interpretation of the facial phenotype, supplementing this analysis with the written descriptions. We also adjusted the nomenclature to reflect the current recommendations according to the Elements of Morphology [34–36], replacing terms for their preferred synonym when appropriate. The one exception was “thin eyebrows” (meaning a decrease in width of eyebrow, subjectively assessed) which has no adequate synonym in the Elements of Morphology.

## Results

### Case reports

#### Patient 1

**Fig. 1:**
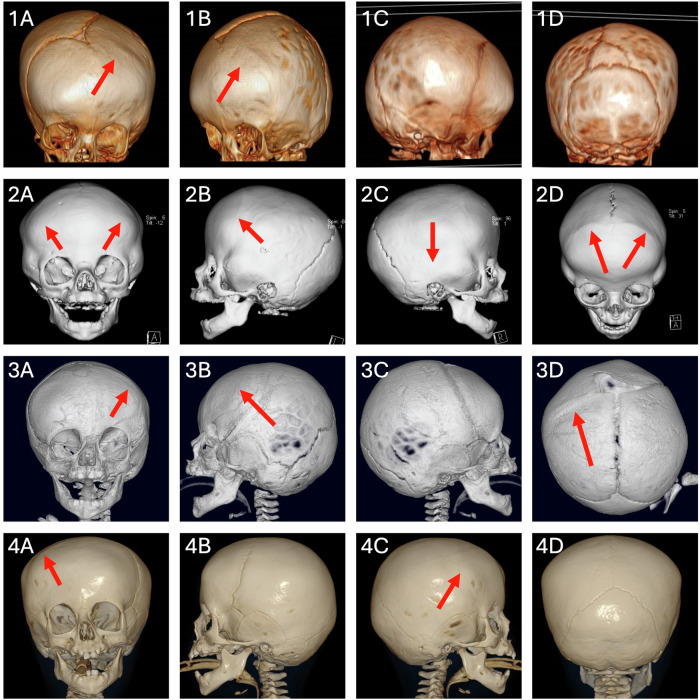
Pre-operative images of 3D CT scans of Patients 1-4. Patient 1 (1**A**–1**D**), Patient 2 (2**A**–2**D**), Patient 3 (3**A**–3**D**) and Patient 4 (4**A**–4**D**). The red arrows indicate prematurely fused sutures.

Patient 1 is a 9 yr 5 mo old female of white British ethnicity. She presented with left unicoronal synostosis (Fig. 1), without symptoms or signs of raised intracranial pressure, and underwent fronto-orbital advancement and remodelling (FOAR) surgery aged 1 yr 8 mo. She also presented with facial dysmorphism (Table 1), moderate intellectual disability, learning difficulties and developmental delay, with receptive, expressive and articulation difficulties in speech, requiring time to process and retrieve words (requiring ongoing speech therapy). She had astigmatism and her only dental anomalies were caries in several teeth. Immune system abnormalities, feeding difficulties, hirsutism, seizures, or dermal problems were absent.

Following standard genetic investigations, the family was enrolled into the Genomics England (GE) 100,000 Genomes Project and trio GS was performed [37], which did not yield clinically reportable variants. The data were subsequently analysed in the GE Research Environment as described [4] and a frameshifting duplication of 28 nucleotides (c.781_808dup; p.(Gly270Alafs*256)) was detected in exon 4 of *BCL11B* (NM_138576.4; ENST00000357195.8), in a small number of reads (4/39). The low proportion of affected reads, further magnified by an alignment problem (the variant was mapped in two ways) explained why the variant had initially been overlooked. Deep sequencing of tissues confirmed the mosaic heterozygous frameshift variant, (Supplementary Material F1–F3).

#### Patient 2

**Fig. 2:**
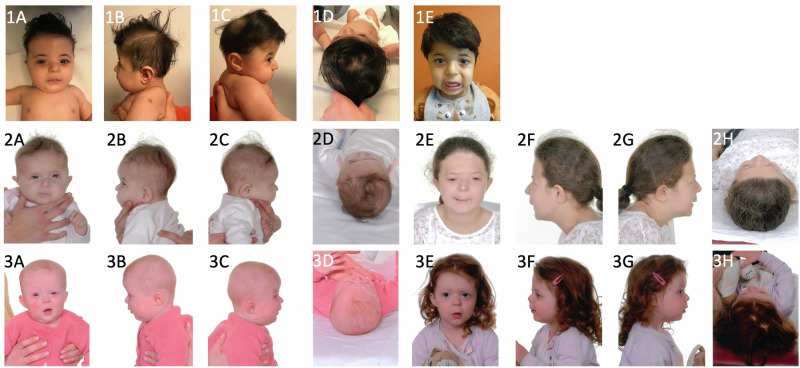
Facial phenotype of Patients 2, 3 and 4. 1) Patient 2 at 12 months (1**A**–1**D**) and 3 years (1**E**). Note thin eyebrows, long/smooth philtrum and thin upper lip vermilion; 2) Patient 3 at 3 months (2**A**–2**D**) and 11 years (2**E**–2**H**). Facial features include asymmetry, thin palpebral fissures, thin/sparse eyebrows, long/smooth philtrum, and thin upper lip vermilion; 3) Patient 4 at 6 months (3**A**–3**D**) and 3 years (3**E**–3**H**). The phenotype comprises facial asymmetry, thin/sparse eyebrows, depressed nasal bridge, long/smooth philtrum, thin upper lip vermilion.

Patient 2 is a 3 yr 4 mo old male of Turkish ethnicity. He presented with CRS of the right squamosal and both coronal sutures. FOAR was performed at age 6 mo. The patient was micro-brachycephalic and presented with mild facial dysmorphism (Fig. 2), delayed motor milestones (head support at 2 mo, sitting without support at 12 mo, walking at 24 mo), delayed speech (first words at 15 mo, 2-word sentences at 42 mo), mild intellectual disability and short attention span.

The patient had recurrent upper respiratory tract infections since infancy (monthly, especially in autumn and winter, lasting 10–14 days). No hospitalization was required. Laboratory analysis revealed eosinophilia and elevated T helper central memory cell count. He also had multiple food allergies and chronic eczema since infancy. Dental anomalies, refractive error, feeding difficulties, hirsutism or seizures were absent. Brain magnetic resonance imaging (MRI) was normal.

Following the clinical assessment, the family had ES as a trio, which detected a de novo frameshift variant in *BCL11B* (NM_138576.4: c.2438_2459del; p.(Val813Alafs*24)). Dideoxy-sequencing confirmed the variant (Supplementary Material F4).

#### Patient 3

Patient 3 is a 14 yr 6 mo female of white British ethnicity. She presented with unicoronal CRS and underwent FOAR at 1 yr 2 mo. Her clinical presentation included facial dysmorphism (Fig. 2), developmental delay and intellectual disability - aged 11 years she produced work 5–6 years below her level, with a full-time Education, Health and Care Plan (EHCP) at a mainstream school. She also had communication difficulties suggestive of autism spectrum disorder and behavioural problems, with a Social Communication Questionnaire (SCQ) score of 18. Other features include bilateral hypermetropia with right astigmatism, a history of feeding difficulties, slow growth, and sleep problems but no symptoms of immune deficiency.

Chromosomal microarray detected a 1.54 Mb microdeletion of chromosome 14q32.2: arr[hg19]14q32.2(99,052,763-100,591,634)x1, encompassing 8 protein-coding genes, including *BCL11B*.

#### Patient 4

Patient 4 is a 4 yr old female of white British ethnicity. She was born after an uncomplicated pregnancy and was noted to have an unusual head shape. She was referred to the craniofacial unit at 6 mo, diagnosed with right unicoronal synostosis and underwent FOAR at 1 yr 4 mo. Of note, her surgery was expedited owing to concerns about increased intracranial pressure affecting development (vocalization stopped for 3–5 mo around the time of surgery). The patient had global developmental delay (sitting with support at 9 mo, walking at 16 mo), intellectual disability (EHCP in place and calls with special educational needs teacher), speech delay (2–3 word phrases at 2 yr 11 mo), facial dysmorphism (Fig. 2) and behavioural issues. Additional features included allergies to egg and peanuts, sleep problems and strabismus with good visual acuity. Immunological workup showed mild abnormalities of lymphocyte subset count (decreased CD19 and CD16/56+ count, increased percentages of CD3 and CD8), without clinical repercussion. Immunoglobulin levels (IgG, IgA and IgM) were normal.

The patient was initially investigated using array-CGH and a 7-gene CRS diagnostic panel, neither of which yielded clinically reportable variants. Subsequent GS of the family trio detected a de novo frameshift variant in *BCL11B* (NM_138576.4:c.400_403dup; p.(Cys135Tyrfs*61). The result was confirmed with Sanger sequencing (Supplementary Material F5).

#### Literature Review

We identified 63 patients from 25 papers, with clinical features attributed to a sequence variant within *BCL11B*. Duplicate patient reports were merged, and cases were excluded if the reported variant did not reach at least likely pathogenic designation upon ACMG reclassification. The final cohort was 51 individuals (48 case reports from 20 papers and 3 individuals from our case series) (Supplementary Table 1) [10–28, 38].

**Fig. 3:**
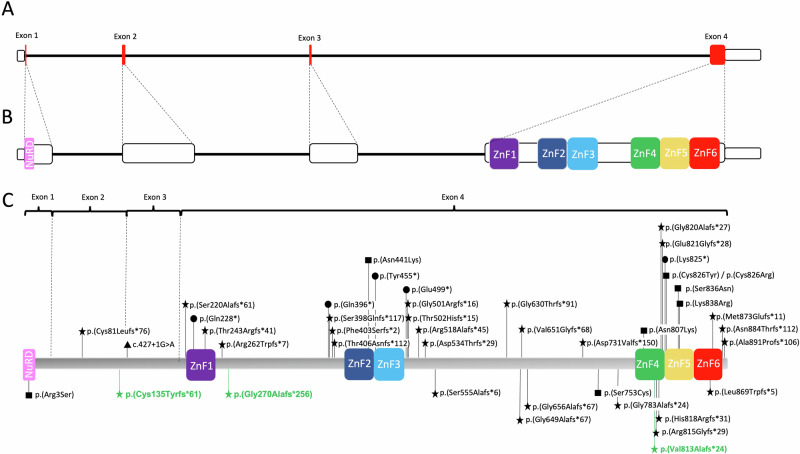
Structure and variants in *BCL11B*. **A** Representation of *BCL11B*. Coding regions are in red. Note the disproportionate size of exon 4. **B** Representation of *BLC11B* with exons out of scale, demonstrating the location of important functional domains (coloured boxes). All the zinc-finger domains are located within exon 4. **C** BCL11B protein (894 amino acids) with the pathogenic variants reported in the literature. Variants below the protein are present in patients with CRS. Variants in green were identified in our cohort. Different shapes represent different variant types (star, frameshift; triangle, splice site; circle, nonsense; square, missense).

Of the 51 individuals heterozygous for *BCL11B* variants, two inherited the variant from an affected mother [11, 20]. In two additional patients, the variant was inherited from a healthy parent with somatic mosaicism [16, 28]. We tabulated 35 distinct truncating variants in 40 patients, including 29 frameshift, one splice site and five nonsense variants (Supplementary Material M3). All but three of these (c.242delG; p.(Cys81Leufs*76), c.400_403dup; p.(Cys135Tyrfs*61), and c.427+1G>A) occurred in exon 4 (Fig. 3), and only two (c.1887_1893del; p. (Gly630Thrfs*91) and c.1944_1965del22; p.(Gly649Alafs*67)), showed recurrence in three [24, 28] and four [11, 14, 28] unrelated individuals, respectively.

In total there were 8 missense variants, of which two showed recurrence: c.1323T>G; p.(Asn441Lys) and c.2421C>G; p.(Asn807Lys), occurring in two [10, 18] and three [11, 14, 19] patients, respectively. Most missense variants are located within important functional domains (zinc fingers or, in one case, the NuRD interacting domain) (Fig. 3), with the exception of (c.2258C>G; p.(Ser753Cys)), which was detected in an individual positive for a recently described *BCL11B*-RD episignature [28].

We compared the clinical features of individuals with missense *BCL11B* substitutions with those of patients with truncating variants. No genotype-phenotype correlations emerged. There was no meaningful way to distinguish between the two OMIM phenotypes associated with *BCL11B*. Therefore, we analysed all the patients as a single cohort.

We analysed facial photographs of all individuals where images were available (36/50), and supplemented this analysis with written facial descriptions. The most recurrent facial features are thin eyebrows in 79% (34/43), thin upper lip vermillion in 89% (39/44), and long philtrum in 73% (33/45). Nose dysmorphism is present in 75.5% of patients, but the specific features are inconsistent, and include low hanging columella (11/45), convex nasal ridge (10/45), wide nasal ridge (8/45) and broad nasal tip (7/45). Blepharophimosis (50%, 22/44), hypertelorism (59.5%, 35.42), sparse eyebrows (53%, 20/38) and smooth philtrum (43%, 19/41) are also common. A composite (Supplementary Material F6) generated from the facial photographs using the asymmetry preserving facial feature point merging approach [39] confirmed these observations.

We identified 12 patients with CRS positive for intragenic mutations (9 from the literature and 3 from our case series); the craniofacial presentation is summarized in Table 2.

**Table 2:** BRD patients with craniosynostosis: craniofacial presentation of patients with sequence variants.

Paper/Patient	Goos (2019)	Patient 4	Patient 1	Pande (2023) Proband 1	Sabbagh (2023) I6	Sabbagh (2023) I3	Sabbagh (2023) I8	Zhao (2022)	Patient 2	Pande (2023) Proband 3	Eto (2022)	Pande (2023) Proband 2
Sex	Male	Female	Female	Female	Female	Male	Male	Male	Male	Male	Male	Female
Age at last examination	19 yr	4 yr	9 yr 5 mo	10 mo	16 yr	11 yr	13 yr	2 yr 1 mo	3 yr 4 mo	5 yr	5 yr	1 yr 1 mo
Variant	c.7C>A; p.(R3S)	c.400_403dup; p.(C135Yfs*61)	c.781_808dup; p.(G270Afs*256)	c.1662_1668del; p.(S555Afs*6)	c.1944_1965del p.(G649Afs*67)	c.1967del p.(G656Afs*67)	c.2258C>G p.(S753C)	c.2346_2361del; p.(G783Afs*24)	c.2438_2459del; p.(V813Afs*24)	c.2443del; p.(R815Gfs*29)	c.2439_2452dup; p.(H818Rfs*31)	c.2605del; p.(L869Wfs*5)
CRS	Bicoronal	Right unicoronal	Left unicoronal	Coronal	N/A	N/A	N/A	Metopic, right coronal	Bicoronal, right squamosal	Sagittal	Sagittal, lambdoid	Sagittal
Surgeries	3	1	1	N/A	N/A	N/A	N/A	N/A	1	N/A	2	N/A
Other features	FD	FD, DD, ID, SI, RA	FD, DD, ID, SI, R, DA	FD, DD, FI, Lab, DA, FP	FD, DD, ID, SI, RA, R, DA	FD, DD, ID, SI, R, DA	FD, DD, ID, SI, FI, RA, DA	FD, DD, ID, SI, Lab, RA, BMRI	FD, DD, ID, SI, FI, Lab, RA	FD, DD, ID, SI, FI, Lab, BMRI	FD, DD, ID, SI, RA, FP	FD, DD, FI, Lab

Patients are listed according to the position of their variants, following a 5′- 3′ orientation. Newly reported cases are listed as Patient 1, Patient 2 and Patient 4.

+ present, − absent, *N/A* information not available, *FD* facial dysmorphism, *DD* developmental delay, *ID* intellectual deficiency, *SI* speech impairment, *FI* frequent or atypical infections, *Lab* laboratory anomalies, *RA* recurrent allergies and/or asthma, *R* refraction errors, *DA* dental anomalies, *FP* feeding problems, *BMRI* brain MRI anomalies.

Synostosis of the coronal suture is relatively more common (67%, 6/9), but sagittal (3/9), lambdoid (1/9) and squamosal (1/9) synostosis have also been reported. One or more surgical interventions may be required. Facial dysmorphism, developmental delay, intellectual disability and speech delay are the most frequently associated features, however, 9 patients also presented with immune system dysregulation.

Additional abnormalities of the cranial skeleton reported in *BCL11B*-related disease include Wormian bones reported in one patient [10] and diastasis of cranial sutures in another [18]. Interestingly both had the same missense variant, c.1323T>G; p.(Asn441Lys).

### The natural history of BRD

Three cardinal clinical features were present in more than 90% of patients: neurodevelopmental disorder (98%, 49/50), characteristic dysmorphic features (98%, 47/48), and immune system dysregulation (93%, 42/45) (Table 3). Patients are usually born at term, after unremarkable pregnancies, although occasional complications have been reported, including maternal hypertension [26] and polyhydramnios [11, 14]. Frequent neonatal complications include hypotonia (observed in at least 11 patients) and feeding problems of varying severity (11/40; 27.5%), often due to difficulty swallowing, which may require oral rehabilitation and/or insertion of a nasogastric or gastrostomy tube (G-tube). These interventions may be temporary, and body weight gain is usually normal despite feeding difficulties, but there are at least two instances of long term G-tube dependency [11, 14].

**Table 3:** Clinical features of BRD.

Cardinal features (present in ≥90% of patients)		
1. Neurodevelopmental disorder	49/50	98%
2. Facial Dysmorphism	47/48	98%
3. Immune dysregulation	42/45	93%
Major features (present in ≥25% of patients)		
• Behavioural anomalies	29/49	59%
• Dental problems	18/44	41%
• Brain MRI anomalies	14/44	32%
• Refraction defects	12/43	28%
• Feeding problems	11/40	27.5%
Minor features (present in ≥15% of patients)		
• Craniosynostosis	12/49	24.5%
• Sleeping problems	11/48	23%
• Hypotonia	11/48	23%
• Constipation and/or reflux disease	10/48	21%
• Autistic features	8/39	20.5%
• Dermatitis and/or eczema	7/48	15%

Developmental delay is a hallmark of BRD, with 96% of patients (47/49) presenting with delayed motor milestones and 94% (45/48) evolving with intellectual disability and/or leaning difficulties. Speech is often impaired (91.5%; 43/47), with speech delay in 87% (41/47) of cases, regression of verbal abilities in one case [20] and dysarthria without early speech delay in another [38]. Age at first words ranged between 12 and 53 months (mean 27.6 mo, median 22 mo, information available for 20/50 cases), but some patients remain non-verbal beyond the 5th year of life [11, 13]. Dysarthria, stuttering, slowing of the verbal processing of information, difficulties with articulation, receptive and expressive language have been documented. Patients may benefit from speech therapy and augmentation devices [11, 27].

Immunodeficiency is present in 93% (42/45) of cases, most frequently as laboratory anomalies (77%; 27/35) without clinical repercussions. In one case, a patient was diagnosed with Severe Combined Immunodeficiency (SCID) before the onset of infections and was treated with allogeneic hematopoietic stem-cell transplantation [10]. Other laboratory anomalies include abnormalities in immunoglobulin levels and in lymphocyte counts on immunophenotyping. Frequent or atypical infections are present in 43.5% of cases (20/46), including haemorrhagic varicella, primary varicella zoster infection, otitis media, skin, urinary and respiratory tract infections. Severity of infection varies from mild, self-limited disease to life threatening infections requiring multiple hospitalisations [26]. Although there is limited information, infections seem to cease after puberty [11]. Allergies and/or asthma are present in 44% of cases (19/43). At least 7 patients have presented with eczema, prurigo nodularis and/or dermatitis, and there have been 4 instances of auto-immune disease [24, 28, 38].

The clinical features of BRD are summarized in Table 3 and Supplementary Material M4. They can be divided into cardinal features (present in ≥90% of patients), major features (present in ≥25% of patients) and minor features (present in ≥15% of patients). The prevalence of minor features and behavioural anomalies may be underestimated, because these were not systematically assessed in the past.

#### Deletions involving BCL11B

**Fig. 4:**
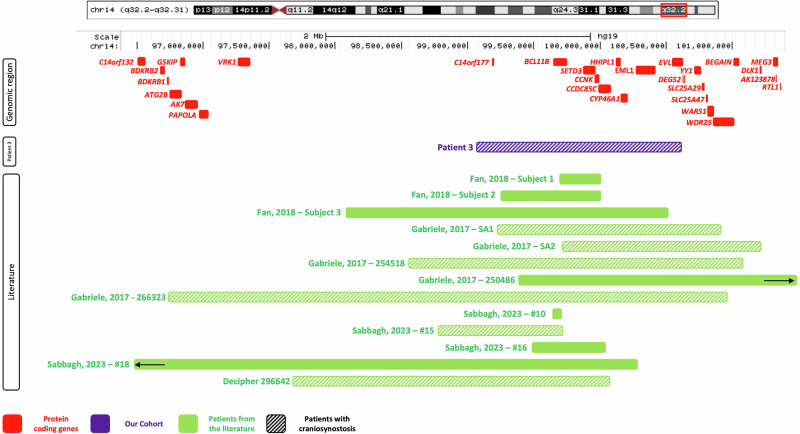
Deletions encompassing *BCL11B.* Genes in the region are represented in red. The deletion identified in Patient 3 is shown in purple. Patients from the literature and/or Decipher are represented in green. Bars with a hatched pattern indicate that CRS was present in that patient. The two black arrows indicate that these deletions extend beyond the picture frame.

Upon identification of Patient 3, we conducted a literature search for individuals with 14q32 microdeletions involving *BCL11B*. This resulted in an additional 12 individuals with deletions of interest [28, 40, 41]. The clinical features of patients with deletions involving *BCL11B* are summarized in Supplementary Table 2. Notably, 50% of the cohort of *BCL11B* deletion patients presented with CRS. We subsequently searched the Decipher database for overlapping deletions within our candidate region for whom CRS was a reported feature and found one additional patient (Fig. 4).

There are 9 cardinal or major features associated with BRD. Seven of those (79%) were observed in the cohort of patients with *BCL11B* deletions (Supplementary Material M5). In contrast, there are 16 features associated with 14q32 terminal microdeletions [33]. Only seven of those (44%) were observed in the *BCL11B* gene deletion cohort, and most (4/7) are also part of BRD (facial dysmorphism, psychomotor delay, hypotonia).

## Discussion

BCL11B is currently associated with two Mendelian phenotypes: IMD49 (OMIM: #617237); and IDDSFTA (OMIM: #618092). The reasons for this split appear to be the severity of the immunodeficiency in the first reported patient and the suggestion of a genotype-phenotype correlation between missense variants and immune dysregulation. Eight years after the original description, no such genotype-phenotype correlation has been established. The initial patient [10] remains the only one affected with SCID. That patient presented with the full triad of cardinal features of BRD, and we are aware of at least one additional individual with the same missense variant, p.(Asn441Lys) who had CD4+ lymphocytopenia, but no clinical repercussions of immunodeficiency [18]. Together these data suggest that immunodeficiency is a feature of BRD (present in more than 90% of patients), rather than a separate entity from the neurodevelopmental phenotype. Furthermore, the severity of neurodevelopmental delay varies from mild to severe, both in patients that harbour missense and those that have truncating variants, thus weakening the initial thought that truncating variants were associated with a milder phenotype.

Regarding IMD49 and IDDSFTA as separate entities dilutes an already limited number of patients, which hinders our understanding of the phenotype, and may have contributed to errors in variant classification in the past. Furthermore, splitting the phenotype of BRD may lead to overlooking immune system manifestations in patients who present primarily with a neurodevelopmental phenotype. In most patients, immune system dysregulation manifests as laboratory anomalies without clinical repercussion, and frequent infections, when present, cease after adolescence [11]. However, at this point, we cannot reach definitive conclusions about its natural history. For this reason, we recommend it be managed similarly to other rare genetic disorders that affect T-cell function: with laboratory assessment immediately after molecular diagnosis (or clinical suspicion), followed by referral to an immunologist if abnormalities are present.

Craniosynostosis is on the threshold of what we consider a major feature of BRD. There is ample functional evidence to corroborate the importance of *BCL11B* in craniofacial development. *BCL11B* is highly expressed in craniofacial suture mesenchyme, and *Bcl11b*-deficient mice develop severe midface hypoplasia and CRS, demonstrating its importance in maintaining patency of sutures [42–45]. Here we described 12 patients heterozygous for *BCL11B* intragenic mutations with CRS. All of them presented with additional features, but CRS was the earliest sign and in one case the most severe element of the presentation [12]. This highlights the importance of investigating molecularly undiagnosed patients with CRS for *BCL11B* pathogenic variants, and reinforces the importance of monitoring the craniofacial development [28].

Until recently the gestalt of BRD was thought of as non-specific. We concluded, however, that BRD has a subtle, but recognizable facial phenotype. The most recurring features are thin and/or sparse eyebrows; thin upper lip vermilion; and long philtrum. At least 85% of patients present with different combinations of these features, and a smaller number of patients (50%) present with a more striking facial phenotype, including bepharophimosis. Important differential diagnoses are fetal alcohol syndrome (blepharophimosis, long philtrum, thin upper lip vermilion, intellectual deficiency) and Ohdo syndrome (OMIM #603736) (blepharophimosis, broad nasal tip, thin upper lip vermilion, hypotonia, feeding problems, delayed motor milestones, speech delay). Additionally, the nose morphology is sometimes reminiscent of 22q11.2 deletion syndrome (OMIM #188400), which may be in the differential diagnosis, especially when immunodeficiency is present.

We described one patient (Patient 3) with a 14q32.2 microdeletion involving *BCL11B*, and reviewed an additional 13 cases from the literature (Supplementary Table 2). Until recently, *BCL11B* had not been recognized as a significant contributor to their clinical phenotype [28, 40, 41], however, there is evidence to suggest that loss-of-function of *BCL11B* is deleterious. The probability of loss-of-function intolerance (pLI) for *BCL11B* is high (0.99), and the loss-of-function observed/expected upper-bound fraction (LOEUF) is 0.28 (highly constrained). Furthermore, functional data suggests that the mechanism through which C-terminally located premature termination codon variants cause BRD is loss-of-function [11].

Because the deletions encompass multiple genes, it is not possible to definitively attribute the clinical features solely to *BCL11B*. For that reason, we studied these patients separately from the cohort we used to delineate the BRD phenotype. Three arguments support the idea that *BCL11B* deletion is a key determinant of pathogenicity. First, their clinical features have more overlap with BRD than with 14q32ter microdeletion syndrome. Second, where facial photographs were available, it was possible to observe that the facial gestalt bears a strong similarity with that of BRD [28, 40]. Third, 50% of patients (7/14) in the *BCL11B* gene deletion cohort presented with CRS, a major malformation that is not usually associated with chromosome 14q32 microdeletions. Four of those individuals harbour deletions that encompass two genes, loss-of-function of which has been associated with CRS: *BCL11B* and *YY1*. Although it is not currently possible to disentangle the relative extent of their contributions to the phenotype (owing to small sample sizes), there is stronger functional evidence supporting the role of *BCL11B* in maintaining the patency of sutures than there is for *YY1* [12, 38, 40, 41].

In summary, major points of interest arising from this study are the delineation of the BRD phenotype and the discussion on the role of 14q32 microdeletions in the molecular pathogenesis of the syndrome. The major limitation was the limited clinical information available. While longitudinal data and a larger number of patients are required to further characterise this syndrome, we believe our work provides an opportunity to better understand BRD, offering insight into diagnostic strategies and disease management.

## Supplementary information

Supplementary Material

Supplementary Table 2

Supplementary Table 1

## Data Availability

Data supporting the findings of this study are available within the article and in Supplemental Material and Supplementary Tables 1–2.
