# Supplementary material for: BCL11B-related disease: a single phenotypic entity?
Source: Eur J Hum Genet. 2025 Mar 3;33(4):451–60. doi: 10.1038/s41431-025-01824-x (PMC11985952; doi:10.1038/s41431-025-01824-x)
Supplement: Supplementary file 1 — Supplementary Material [file 41431_2025_1824_MOESM1_ESM.docx]

**Supplementary Material**

**Supplementary Material M1: Literature search methodology for 14q32 microdeletions involving *BCL11B* (Medline)**

|  | **Literature Search methodology for Medline database** |  |
| --- | --- | --- |
| # | **Search Terms** | **Results** |
| 1 | 14q32*.mp. [mp=title, book title, abstract, original title, name of substance word, heading word, floating sub-heading word, keyword heading word, organism supplementary concept word, protocol supplementary concept word, rare disease supplementary concept word, unique identifier, synonyms, population supplementary concept. word, anatomy supplementary concept word] | 1341 |
| 2 | [deletion.mp](http://deletion.mp/). or Chromosome Deletion/ or Sequence Deletion/ | 250352 |
| 3 | [CNV.mp](http://cnv.mp/). or DNA Copy Number Variations/ or ("copy number varia*" "copy number" or "copy number loss").mp. [mp=title, book title, abstract, original title, name of substance word, heading word, floating sub-heading word, keyword heading word, organism supplementary concept word, protocol supplementary concept word, rare disease supplementary concept word, unique identifier, synonyms, population supplementary concept. word, anatomy supplementary concept word] | 55288 |
| 4 | [BCL11B.mp](http://bcl11b.mp/). | 453 |
| 5 | 1 and 2 | 285 |
| 6 | (1 and 3) not 5 | 70 |
| 7 | 5 or 6 | 355 |
| 8 | (2 and 4) not 7 | 48 |
| 9 | 1. The paper should describe at least one 14132.2 deletion smaller than 5Mb  2. The deletion should involve *BCL11B* partially or completely  3. The paper should contain breakpoint coordinates  4. The Paper should contain clinical information about the individual carrying the deletion. |  |

*Supplementary Material M1*: Literature review methodology for copy number variants involving *BCL11B* (Medline). First, concept A (line #1) was crossed with concept B (lines #2 and #3). Second, concept C (line #4) was crossed with concept B (lines #2 and #3). The resulting 403 entries were screened against the inclusion criteria (line #9).

**Supplementary Material M2: Literature search methodology for 14q32 microdeletions involving *BCL11B (Medline)***

|  | **Literature Search methodology for EMBASE database** |  |
| --- | --- | --- |
| # | **Search Terms** | **Results** |
| 1 | 14q32*.mp. [mp=title, book title, abstract, original title, name of substance word, heading word, floating sub-heading word, keyword heading word, organism supplementary concept word, protocol supplementary concept word, rare disease supplementary concept word, unique identifier, synonyms, population supplementary concept. word, anatomy supplementary concept word] | 1995 |
| 2 | [deletion.mp](http://deletion.mp/). or Chromosome Deletion/ or Sequence Deletion/ or [CNV.mp](http://cnv.mp/). or DNA Copy Number Variations/ or ("copy number varia*" "copy number" or "copy number loss").mp. | 425242 |
| 3 | 1 and 3 | 659 |
| 4 | 1. The paper should describe at least one 14132.2 deletion smaller than 5Mb  2. The deletion should involve *BCL11B* partially or completely  3. The paper should contain breakpoint coordinates  4. The paper should contain clinical information about the individual carrying the deletion. |  |

*Supplementary Material M2*: Literature review methodology for copy number variants involving *BCL11B* (Embase). Concept A (line #1) was crossed with concept B (line #2). The resulting 659 entries were screened against the inclusion criteria listed on line #4.

**Supplementary Material M3: Summary of the number of patients with different *BCL11B* variants**

| Category and type of *BCL11B* variant | No. of Patients | Unique variants |
| --- | --- | --- |
| Sequence variants: | 51 | 43 |
| Frameshift | 34 | 29 |
| Nonsense | 5 | 5 |
| Missense | 11 | 8 |
| Splice site | 1 | 1 |
| 14q32.2 microdeletions | 14 | 14 |
| Total | 65 | 57 |

**Supplementary Material M4: Breakdown of cardinal features of BRD**

| Cardinal features (present in ≥ 90% of patients) |  |  |
| --- | --- | --- |
| 1. Neurodevelopmental disorder | 49/50 | 98% |
| - Delayed motor milestones | 47/49 | 96% |
| - Intellectual deficiency | 45/48 | 94% |
| - Speech impairment | 43/47 | 91.5% |
| 1. Facial Dysmorphism | 47/48 | 98% |
| - Thin upper lip vermilion | 39/44 | 89% |
| - Thin and/or sparse eyebrows | 36/44 | 82% |
| - - Thin eyebrows | 34/43 | 79% |
| - - Sparse eyebrows | 20/38 | 53% |
| - Long and/or smooth philtrum | 36/45 | 80% |
| - - Long Philtrum | 33/45 | 73% |
| - - Smooth philtrum | 19/41 | 43% |
| - Hypertelorism | 25/42 | 59.5% |
| - Blepharophimosis | 22/44 | 50% |
| 1. Immune dysregulation | 42/45 | 93% |
| - Frequent and/or atypical infections | 20/46 | 43.5% |
| - Laboratory anomalies | 27/35 | 77% |
| - Recurrent allergies and/or asthma | 19/43 | 44% |

**Supplementary Material M5: Phenotypic overlaps between *BCL11B* gene deletions and BRD or 14q32-qter microdeletions**

| Features of 14q32-qter microdeletions | Features of BRD |
| --- | --- |
| Oligohydramnios  Premature birth  **Failure to thrive**  **Facial dysmorphism**  **Psychomotor delay**  **Language delay**  **Hypotonia**  Seizures  Weak cry  **Microcephaly**  GE reflux  Nystagmus  SN hearing loss  Small hands/feet  Bicuspid aortic valve  **Small for gestational age** | Cardinal Features |
|  | **Neurodevelopmental disorder***  **Facial dysmorphism***  **Immune dysregulation*** |
|  | Major Features |
|  | Behavioural anomalies  Dental problems  **Brain MRI anomalies**  **Refraction errors**  **Feeding difficulties**  **Craniosynostosis** |
|  | Minor Features |
|  | **Sleeping problems** **Hypotonia** **Constipation and/or reflux disease** **Autistic features** Dermatitis and/or eczema |

Features marked in bold are present in patients with 14q32.2 interstitial deletions involving *BCL11B*.

**Supplementary Material F1**

*Supplementary Material F1*: IGV screengrab of WGS analysis from the 100,000 Genomes Project showing evidence of the insertion in the proband (red arrows), which is absent in both parents (from top to bottom: proband, mother and father).


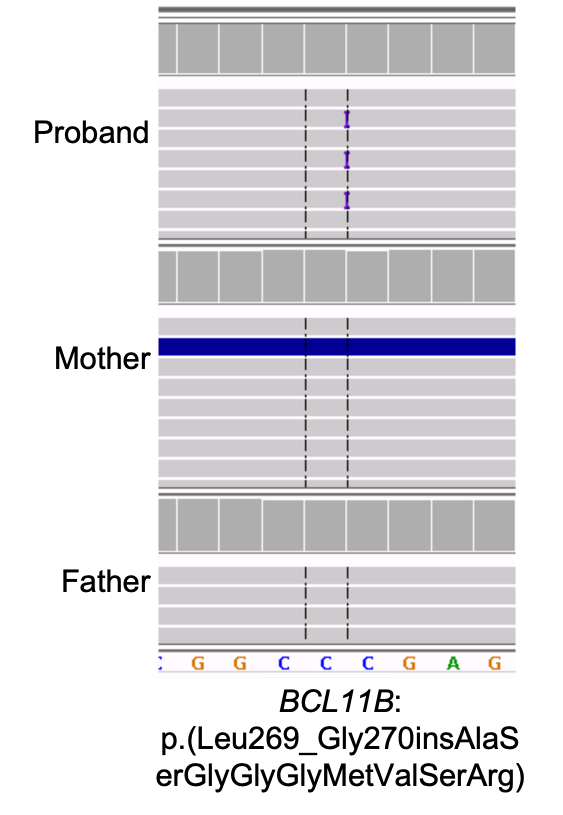


**Supplementary Material F2**


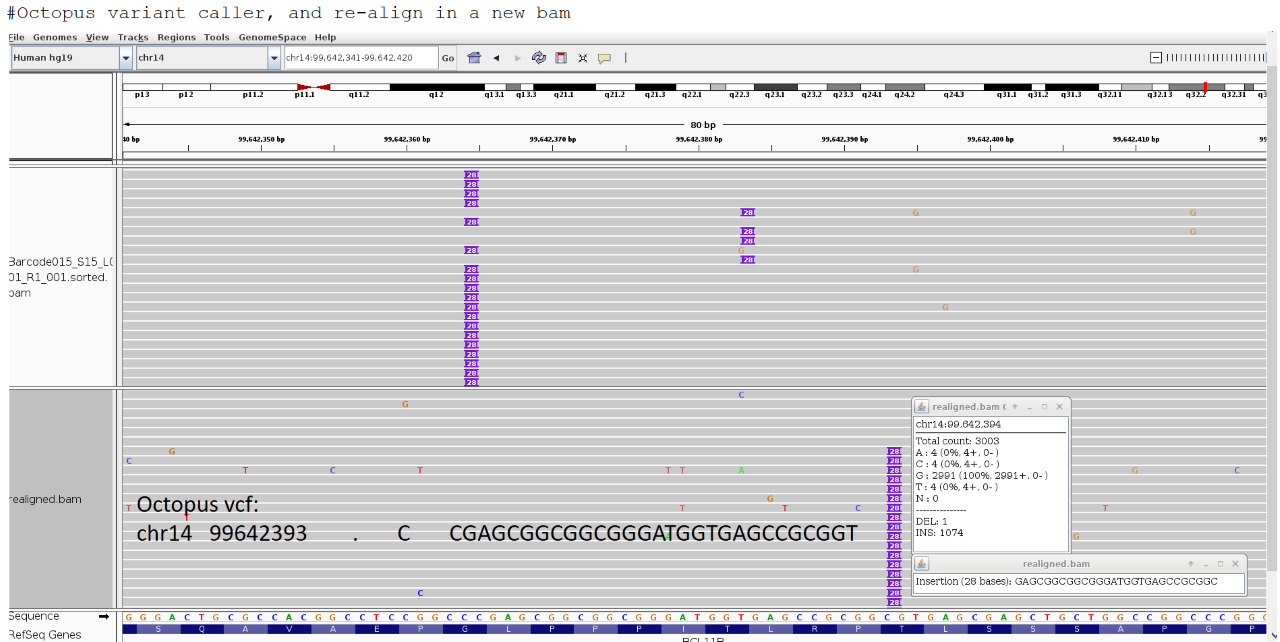


*Supplementary Material F2*: IGV screengrab of the insertion after resequencing. Note that the 28 bp insertion has been mapped/annotated in 2 different ways, lowering the apparent proportion of each variant call to ~13%, which led to the variant being filtered out in the initial analysis.

**Supplementary Material F3**


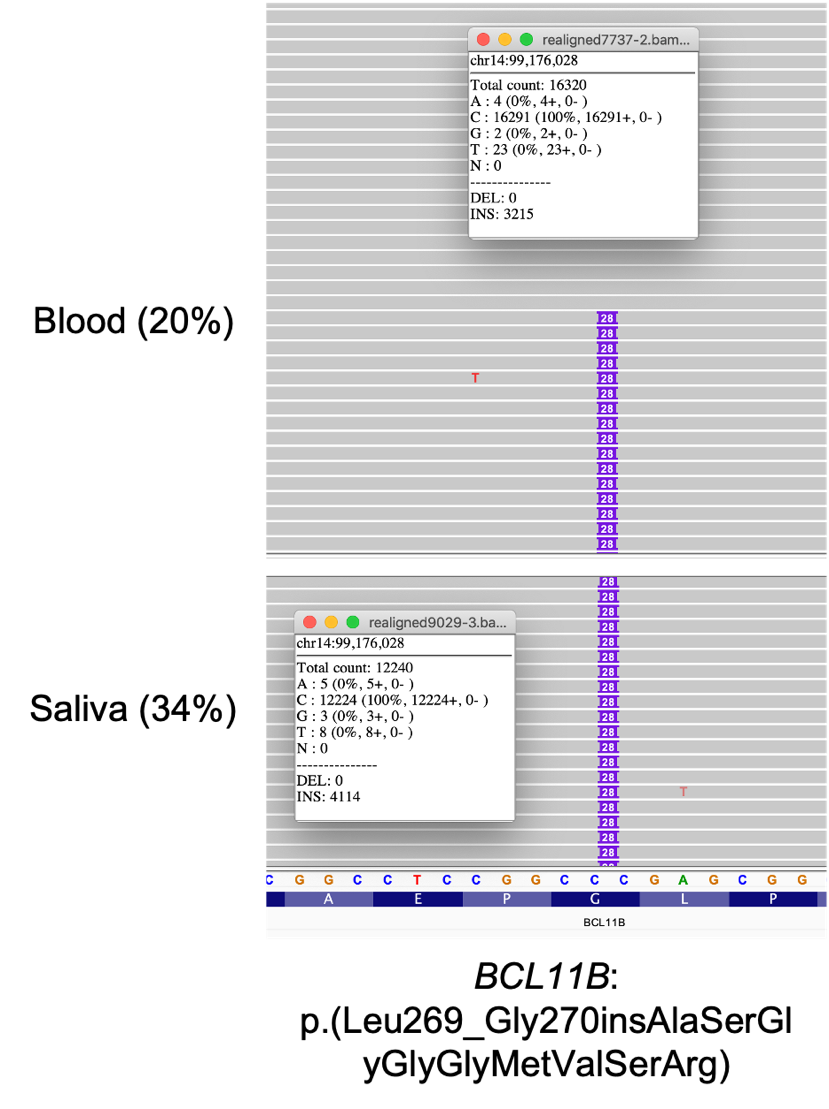


*Supplementary Material F3*: IGV screengrab of deep sequencing of blood and saliva, showing the number of reads containing the insertion (inset boxes; ins, insertion). Insertion read depth indicates a total of 20% of mutated reads in blood (3215/16320) and 34% in saliva (4114/12240).

**Supplementary Material F4**


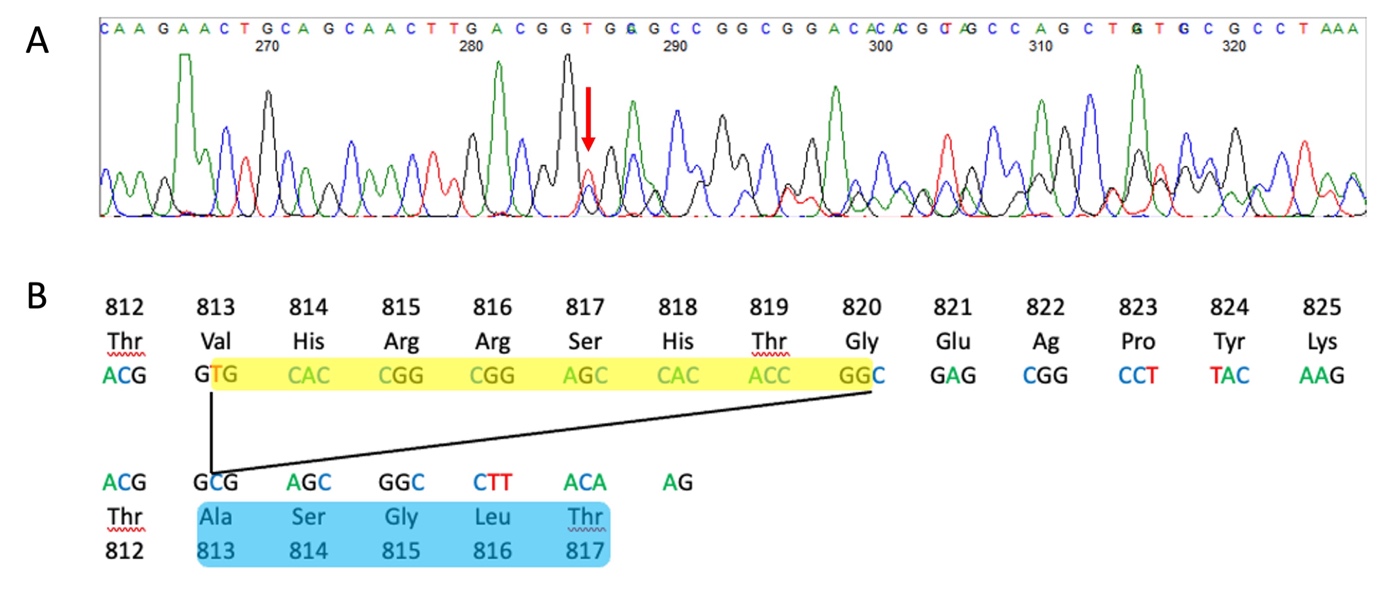


*Supplementary Material F4*: A) Dideoxy-sequencing confirmed the deletion of 22 bases in Patient 2 (c.2438_2459del;p.(Val813Alafs*24)) and its absence in the parents (not shown). The red arrow marks the beginning of the deletion. B) Nucleotide and amino acid sequence around the deletion. Deleted bases are highlighted in yellow, and the novel sequence is highlighted in blue.

**Supplementary Material F5**


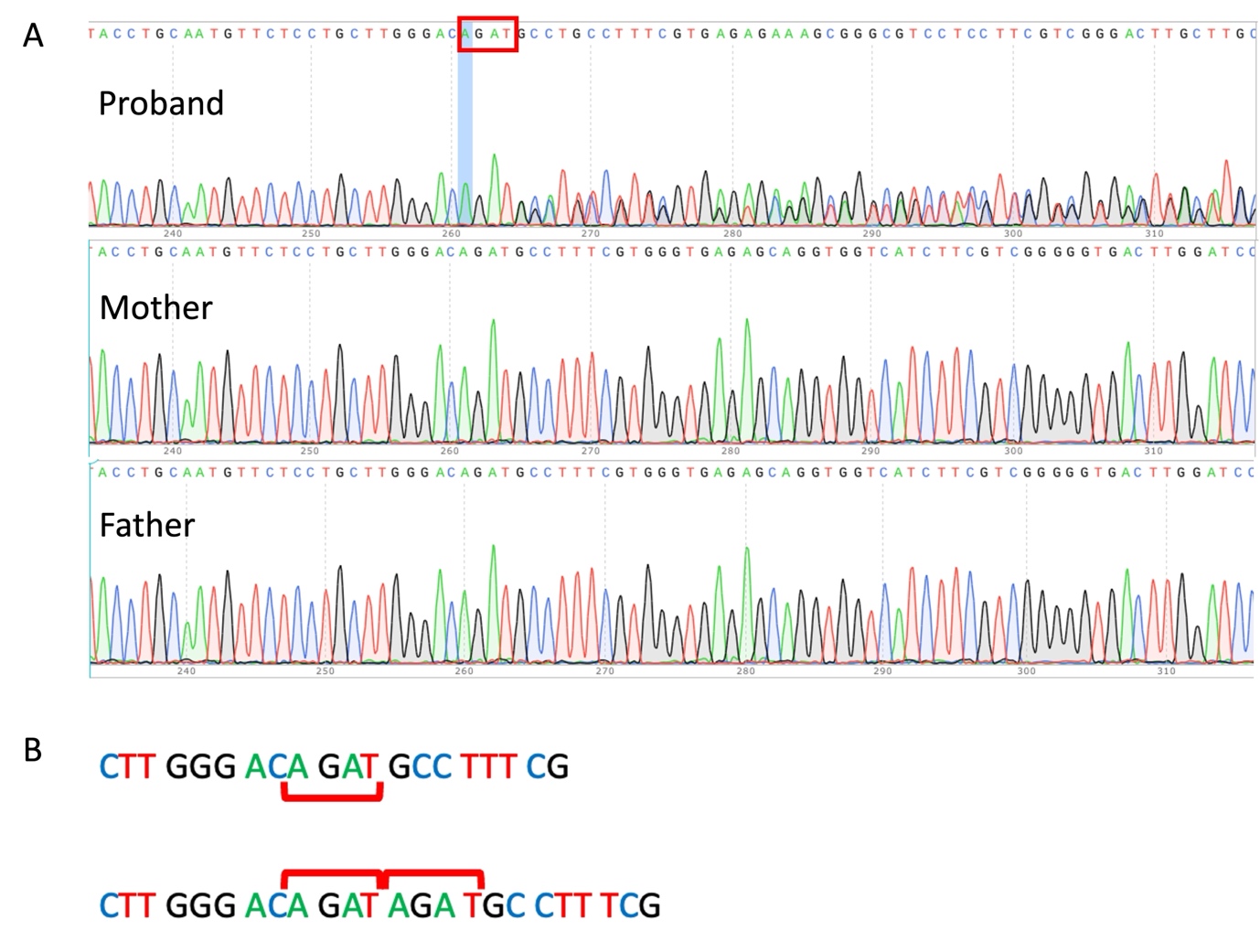


*Supplementary Material F5*: A) Dideoxy-sequencing confirmed the variant in Patient 4 (c.400_403dup) and its absence in the parents*.* The duplicated bases are indicated by the red box. B) Sequence at the duplication. The upper line shows the normal sequence (duplicated bases highlighted in yellow) and the lower line shows the new sequence after duplication. The red brackets mark the duplicated bases.

**Supplementary Material F6**


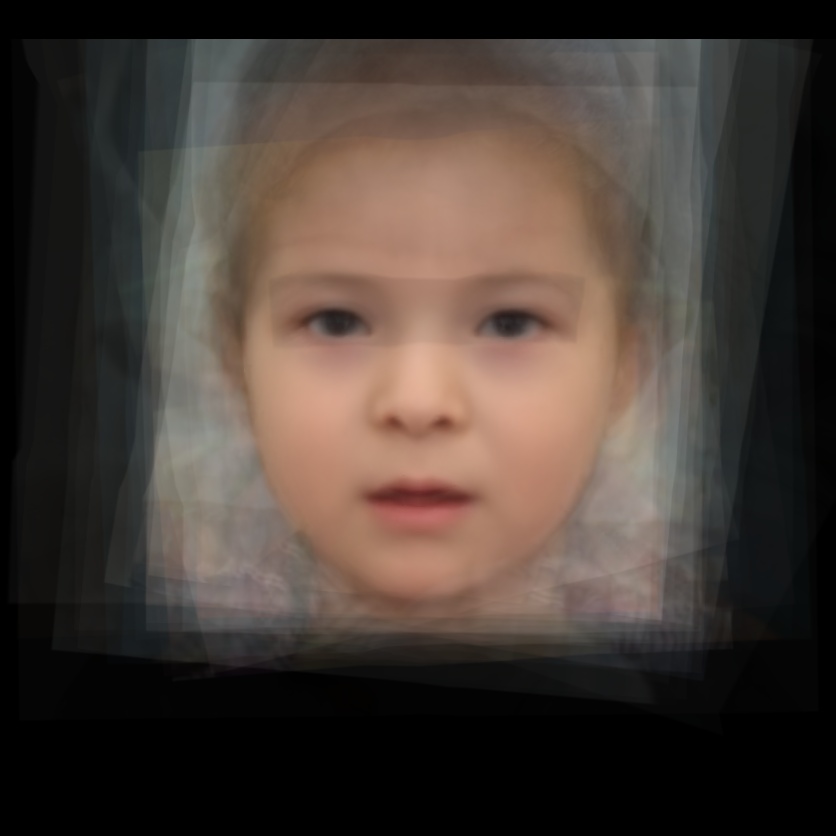


*Supplementary Material F6*: Composite of the facial appearance of patients with BRD. Note the sparse eyebrows, thin upper lip vermillion and long philtrum.
